# The heterogeneous human memory CCR6+ T helper-17 populations differ in T-bet and cytokine expression but all activate synovial fibroblasts in an IFNγ-independent manner

**DOI:** 10.1186/s13075-021-02532-9

**Published:** 2021-06-03

**Authors:** Wendy Dankers, Hannah den Braanker, Sandra M. J. Paulissen, Jan Piet van Hamburg, Nadine Davelaar, Edgar M. Colin, Erik Lubberts

**Affiliations:** 1 Department of Rheumatology, Erasmus MC, University Medical Center Rotterdam, Dr. Molewaterplein 40, 3015 GD Rotterdam, The Netherlands; 2 Department of Immunology, Erasmus MC, Rotterdam, The Netherlands; 3 Department of Internal Medicine, Erasmus MC, Rotterdam, The Netherlands; 4 Current address: Rheumatology Research Group, Centre for Inflammatory Diseases, School of Clinical Sciences at Monash Health, Monash University, Clayton, Australia; 5 Current address: Amsterdam Rheumatology and Immunology Center, Department of Clinical Immunology & Rheumatology and Laboratory for Experimental Immunology, Academic Medical Center/University of Amsterdam, Amsterdam, The Netherlands

**Keywords:** Inflammatory arthritis, Cytokines, T cells, Th17, Th17.1, Vitamin D, Synovial fibroblasts, Transcription factors

## Abstract

**Background:**

Chronic synovial inflammation is an important hallmark of inflammatory arthritis, but the cells and mechanisms involved are incompletely understood. Previously, we have shown that CCR6+ memory T-helper (memTh) cells and synovial fibroblasts (SF) activate each other in a pro-inflammatory feedforward loop, which potentially drives persistent synovial inflammation in inflammatory arthritis. However, the CCR6+ memTh cells are a heterogeneous population, containing Th17/Th22 and Th17.1 cells. Currently, it is unclear which of these subpopulations drive SF activation and how they should be targeted. In this study, we examined the individual contribution of these CCR6+ memTh subpopulations to SF activation and examined ways to regulate their function.

**Methods:**

Th17/Th22 (CXCR3^−^CCR4^+^), Th17.1 (CXCR3^+^CCR4^−^), DP (CXCR3^+^CCR4^+^), and DN (CXCR3^−^CCR4^−^) CCR6+ memTh, cells sorted from PBMC of healthy donors or treatment-naïve early rheumatoid arthritis (RA) patients, were cocultured with SF from RA patients with or without anti-IL17A, anti-IFNγ, or 1,25(OH)_2_D_3_. Cultures were analyzed by RT-PCR, ELISA, or flow cytometry.

**Results:**

Th17/Th22, Th17.1, DP, and DN cells equally express *RORC* but differ in production of *TBX21* and cytokines like IL-17A and IFNγ. Despite these differences, all the individual CCR6+ memTh subpopulations, both from healthy individuals and RA patients, were more potent in activating SF than the classical Th1 cells. SF activation was partially inhibited by blocking IL-17A, but not by inhibiting IFNγ or *TBX21*. However, active vitamin D inhibited the pathogenicity of all subpopulations leading to suppression of SF activation.

**Conclusions:**

Human CCR6+ memTh cells contain several subpopulations that equally express *RORC* but differ in *TBX21*, IFNγ, and IL-17A expression. All individual Th17 subpopulations are more potent in activating SF than classical Th1 cells in an IFNγ-independent manner. Furthermore, our data suggest that IL-17A is not dominant in this T cell-SF activation loop but that a multiple T cell cytokine inhibitor, such as 1,25(OH)_2_D_3_, is able to suppress CCR6+ memTh subpopulation-driven SF activation.

**Supplementary Information:**

The online version contains supplementary material available at 10.1186/s13075-021-02532-9.

## Background

Persistent synovial inflammation is a hallmark of inflammatory arthritis such as psoriatic arthritis (PsA) and rheumatoid arthritis (RA). This results in pain, fatigue, and functional disability, which cannot be sufficiently suppressed in a large group of patients due to ineffective treatment response or treatment resistance [1–3]. In order to further improve the quality of life of these patients, it is important to understand the underlying pathogenesis and which cells play a role in driving the chronic inflammation.

The relevance of T cell biology in inflammatory arthritis is supported by their infiltration into arthritic joints and also by various RA associated genes, including MHC class II genes, *PTPN22, CTLA-4,* and *CCR6*, that are related to T cell biology [4–6]. Both Th1 and Th17 cells are present in inflammatory arthritis, although which of these cell types drives chronicity of joint inflammation is not fully elucidated [7]. Moreover, with the discovery of the newly identified Th17.1 cells, which express *TBX21* and produce IFNγ but are also *RORC* positive, the potential role for IFNγ-producing Th1 cells in arthritis needs to be reconsidered [8, 9].

Importantly, both Th17 and Th17.1 cells express the chemokine receptor CCR6. Previously, we and others have shown that CCR6+ memory T-helper (memTh) cells are elevated in the blood of treatment-naïve early RA patients and are present in the synovial fluid [10, 11]. These cells express the transcription factor *RORC* and pro-inflammatory cytokines such as interleukin (IL)-17A, tumor necrosis factor alpha (TNFα), and interferon-gamma (IFNγ) [12]. Functionally, they contribute to synovial inflammation by activating synovial fibroblasts (SF), which in turn further activate the CCR6+ memTh cells to create a pro-inflammatory loop. This loop also leads to the induction of IL-8 to attract more immune cells, of prostaglandin-E2 (PGE2), IL-6, and IL-1β to activate other immune cells and of matrix metalloproteases (MMPs) that contribute to the tissue damage [10].

However, CCR6+ memTh cells are a heterogeneous group of cells which can be further subdivided based on their expression of the chemokine receptors CCR4 and CXCR3 [12]. Th17/Th22 cells, distinguished as CCR4+ CXCR3- CCR6+ memTh cells, express high levels of IL-17A and (very) low to absent IFNγ, and are *RORC* and IL-22 positive. Double-positive (DP) cells, expressing both CCR4 and CXCR3, produce both IL-17A and IFNγ and are *RORC* and *TBX21* positive. Th17.1 cells, or also called non-classical Th1 cells, are identified as CCR4- CXCR3+ CCR6+ memTh cells and co-express *RORC* and *TBX21*, but also high levels of IFNγ and low amounts of IL-17A. Th17.1 cells are specifically elevated at the inflammatory sites in autoimmune diseases such as sarcoidosis, Crohn’s disease, and juvenile idiopathic arthritis [9, 13, 14]. Interestingly, there is also a subpopulation within CCR6+ memTh cells that is less well described. They express neither CCR4 nor CXCR3 receptors (double-negative cells, DN) [12].

Although the CCR6+ memTh are found at inflammatory sites, it is unclear which of the subpopulations is most capable of initiating synovial inflammation. Classically, Th17 cells were thought to be the main drivers of chronic joint inflammation leading to tissue destruction through their production of IL-17A [10]. However, we previously described that Th17.1 and DP cells are elevated in ACPA positive RA patients compared to ACPA negative patients, suggesting a distinct role for these cells in different RA subtypes [15]. Also in other immune diseases, such as Crohn’s disease and multiple sclerosis, Th17.1 cells are now considered to be more pathogenic [9, 16]. The role of DN cells is not clear, since it has not been previously studied in arthritis.

Further characterization and understanding of which of the CCR6+ memTh subpopulations are predominantly responsible for driving synovial fibroblast activation could aid in developing more targeted therapies for inflammatory arthritis. Therefore, in this study, we examined the individual contribution of these CCR6+ memTh subpopulations to SF activation and examined ways to regulate their function.

## Methods

### Subjects

Peripheral blood mononuclear cells (PBMC) from 23 healthy individuals were isolated from buffycoats (Sanquin Bloodbank, Rotterdam, the Netherlands). RA PBMC were isolated from 41 treatment-naïve early RA patients embedded in the Treatment in the Rotterdam Early Arthritic Cohort (tREACH), which all fulfilled the American College of Rheumatology 2010 classification criteria for RA. The study was approved by the Medical Ethics Review Board of Erasmus MC Rotterdam. Synovial fibroblasts were isolated from established RA patients undergoing joint replacement surgery. Relevant clinical characteristics are shown in Additional file 1 (treatment-naive early RA patients) and Additional file 2 (established RA patients). Informed consent was obtained from all individuals.

### Cell sorting

PBMC were isolated from peripheral blood or buffycoats through a ficoll gradient and stored in liquid nitrogen until use. For sorting, isolated PBMC were first pre-purified for CD4 using magnetic beads (Miltenyi Biotec) and then sorted using monoclonal antibodies against CD4, CD25, CXCR3, CCR4 (BioLegend, San Diego, CA, USA), CD45RO, and CCR6 (BD Biosciences, San Diego, CA, USA). All cells were CD4^+^CD45RO^+^CD25^low/int^, and then further sorted on CCR6^−^CCR4^−^CXCR3^+^ (Th1), CCR6^+^CCR4^+^CXCR3^−^ (Th17/Th22), CCR6^+^CCR4^−^CXCR3^+^ (Th17.1), CCR6^+^CCR4^+^CXCR3^+^ (DP), or CCR6^+^CCR4^−^CXCR3^−^ (DN).

### Cell culture

RASF were grown out of small biopsies from synovial tissue after joint replacement surgery. The cells were cultured in Dulbecco’s modified Eagle medium (DMEM), supplemented with 10% fetal calf serum (FCS, Gibco, Waltham, MA, USA), and 100 U/ml penicillin/streptomycin (pen/strep, Lonza, Verviers, Belgium) and used between passage 3 and 8.

Sorted CCR6+ memTh subpopulations were cultured at a density of 2.5 × 10^4^ cells/ml in Iscove’s modified Dulbecco’s medium (IMDM) supplemented with 10% FCS, 2 mM L-glutamine (Lonza), 100 U/ml pen/strep, and 50 μM β-mercaptoethanol (Sigma-Aldrich, St. Louis, MO, USA). Where indicated, the CCR6+ Th memory subpopulations were cocultured with 1.0 × 10^4^ RA synovial fibroblasts (RASF), which were seeded in flat-bottom 96-well plates 24 h earlier. T cells were stimulated with 300 ng/ml soluble anti-CD3 and 400 ng/ml soluble anti-CD28 (Sanquin, Amsterdam, the Netherlands). For some experiments, the cells were cultured with 100 nM 1,25(OH)_2_D_3_ dissolved in 100% ethanol (Leo Pharmaceutical Products, Ballerup, Denmark), 100 μg/ml secukinumab (anti-IL-17A, Novartis, Basel, Switzerland), or 10 μg/ml anti-IFNγ (R&D Systems, Minneapolis, MN, USA). Controls were an equal volume of 100% ethanol (with a maximum final ethanol concentration of 0,1%) or equal concentrations of corresponding isotype control antibodies, respectively.

### Lentiviral vectors and T cell transduction

LVRU6MP plasmids containing scrambled short hairpin RNA (shRNA) or four different *TBX21*-targeting shRNAs were obtained from GeneCopoeia (Rockville, MD, USA). They were amplified in GCI-5α competent cells (GeneCopoeia) and extracted using the PureYield Plasmid Midiprep System (Promega Benelux, Leiden, the Netherlands), both following manufacturer’s protocol. LVRU6MP plasmids and the Lenti-PAC HIV Expression Packaging Kit (GeneCopoeia) were used to generate replication-deficient lentiviral vectors in 293T cells (GeneCopoeia) as described in manufacturer’s protocol. 293T culture supernatant containing lentiviral vectors was filtered through 0.45 μm polyethersulfone filters and stored at – 80 °C until use.

For transduction of T cells, CCR6+ Th memory cells (CD4^+^CD45RO^+^CCR6^+^CD25^lo/int^) were sorted and activated with 300 ng/ml soluble anti-CD3 and 400 ng/ml soluble anti-CD28 for 2 days. Then, medium was replaced with either 50% 293T culture supernatant containing relevant lentiviral vectors and 50% normal T cells medium, or 100% T cell medium for mock conditions, both supplemented with 8 ng/ml polybrene (Sigma-Aldrich). T cells were then transduced by centrifuging cells at 1000×*g* and 32 °C for 90 min, followed by 4 h incubation at 37 °C and 5% CO_2_. Then, transduction medium was removed and cells were further cultured in normal T cell medium at a density of ~ 0.1 × 10^4^ cells/ml together with 0.5 × 10^5^ autologous irradiated PBMC (50 Gy, using RS320, X-strahl, Surrey, UK) and 50 ng/ml phytohaemaglutinin (PHA). After 6 days of culture, cells were restimulated with 5 ng/ml IL-2 and cultured for another 6 days. Then, the stably transduced cells were sorted based on mCherry expression and cultured with SF as described in the cell culture section.

### Flow cytometry

For intracellular cytokine detection by flow cytometry, cultured cells were restimulated for 4 h using 50 ng/ml phorbol 12-myristate 13 acetate (PMA) (Sigma-Aldrich), 500 ng/ml ionomycin (Sigma-Aldrich), and Golgistop (BD Biosciences). Restimulated cells were stained with Fixable Viability Dye eFluor506 (eBioscience, San Diego, CA, USA) following manufacturer’s instructions. For intracellular staining, the cells were fixed with 2% paraformaldehyde in PBS and permeabilized using 0.5% saponin in FACS buffer (PBS with 0.5% BSA and 0.05% NaN3). Monoclonal antibodies against IL-17A, IL-22 (eBioscience), and IFNγ (BioLegend) were then used for staining in permeabilization buffer. Samples were measured on a FACSCantoII Flow Cytometer (BD Biosciences).

### ELISA

Concentration of IL-17A, IL-17F, IL-22, IFNγ, GM-CSF, IL-6, IL-8 (all eBioscience), MMP1, and MMP3 (both R&D systems) were measured in culture supernatant using ELISA following manufacturer’s protocols.

### RT-PCR

RNA was isolated from the (co)cultured cell lysates with the GenElute Mammalian Total RNA Miniprep Kit (Sigma-Aldrich), treated with 0.1 U/μl DNAse (Invitrogen) and reverse transcribed into cDNA using 10 U/μl Superscript II (Invitrogen) and random hexamer primers. The PCR was performed using TaqMan Universal Mastermix (Thermofisher Waltham, Massachusetts, USA). Primers and probes were designed and chosen using ProbeFinder Software and the Universal Probe library (Roche Applied Science, Indianapolis, IN, USA) and are listed in Additional file 3. The qPCR reaction per gene included 10 ng μl of cDNA, 1x l Taqman Universal Mastermix, 10 pmol forward primer, 10 pmol reverse primer, and 0.012 μM probe. The RT-PCR was performed with the Viia7 System (Applied Biosystems, Waltham, MA, USA), with an initial cycle at 50 °C for 2 min and a heating cycle at 95 °C for 10 min, followed by 45 cycles of 30 s at 95 °C and 30 s at 60 °C. Hypoxanthine phosphoribosyltransferase (*HPRT*) was used to normalize transcript levels. RT-PCR was performed with the Viia7 System and analyzed using QuantStudio Real-Time PCR Software version 1.3 (Applied Biosystems).

### Statistical analysis

To test differences between two or more groups with equal variances, Student’s T-test or ANOVA with a Tukey post-test were used, respectively. For groups with unequal variances, Kruskal-Wallis tests were performed with Dunn post-test. For the effectives of the shRNA-mediated knockdown of *TBX21,* we used one-sample t-tests. Statistical analyses were done using Prism software version 6.01 (GraphPad Software, La Jolla, CA, USA).

## Results

### CCR6+ memTh subpopulations differ in cytokine production, but not in expression of *RORC*, in healthy donors and treatment-naïve RA patients

**Fig. 1:**
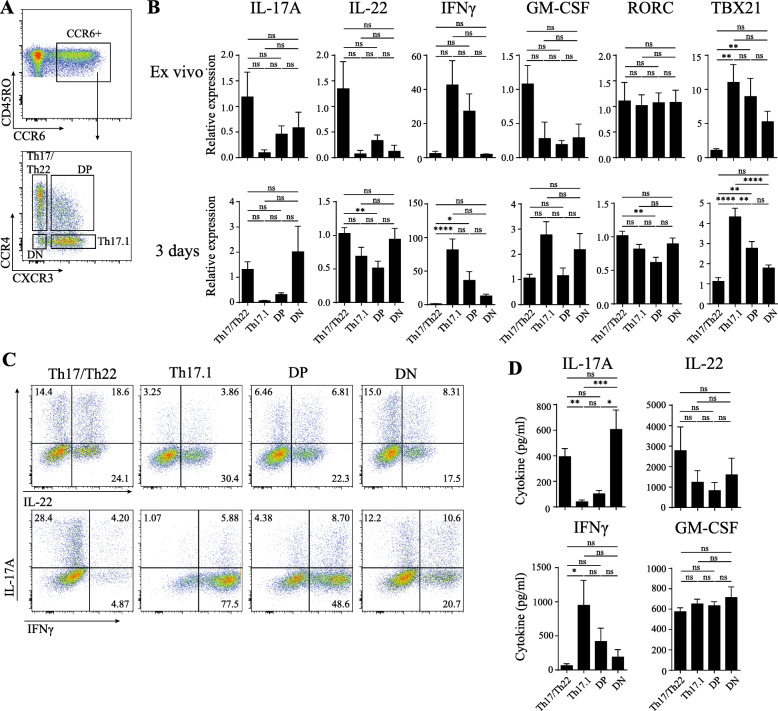
CCR6+ Th memory subpopulations differ in cytokine production, but not in *RORC* expression. **a** Gating strategy for the four subpopulations. **b** Gene transcription levels of sorted cells, directly ex vivo (upper panel) or after 3 days of stimulation with anti-CD3 and anti-CD28 (lower panel) is determined using RT-PCR. **c** Representative flow cytometry plot of IL-17A, IL-22, and IFNγ expression after 3 days of stimulation from one healthy donor. **d** Production of IL-17A, IL-22, IFNγ, and GM-CSF after 3 days of stimulation with anti-CD3 and anti-CD28 as measured by ELISA. Data represent mean ± SEM of n = 3–8 healthy controls of at least 2 independent experiments. *p < 0.05, **p < 0.01, ***p < 0.001, ****p < 0.0001

For studying the role and properties of CCR6+ memTh subpopulations, Th17/Th22, Th17.1, double-positive (DP), and double-negative (DN) cells were sorted from healthy PBMC based on the chemokine receptors CXCR3 and CCR4 (Fig. 1A, Additional file 4). Directly after sorting, Th17/Th22 cells showed a trend towards being the strongest producers of IL-17A, IL-22, and GM-CSF, whereas Th17.1 and DP cells produced the highest levels of IFNγ (Fig. 1B, upper panel). Furthermore, Th17.1 and DP cells produced significantly more *TBX21* than Th17/Th22 cells. Interestingly, *RORC* was equal in all subpopulations despite the differences in IL-17A expression (Fig. 1B, upper panel).

Since the phenotype stability of the subpopulations is currently unknown, these sorted cells were cultured for three days under stimulation of anti-CD3 and anti-CD28 and examined for cytokine and transcription factor expression (Fig. 1B, lower panel). After 3 days, both Th17/Th22 and DN cells expressed high levels of IL-17A mRNA, although the differences with Th17.1 and DP cells did not reach statistical significance. All subpopulations expressed IL-22, with DP cells producing slightly less than the Th17/Th22 population. After 3 days of culture, Th17.1 and DP cells expressed significantly more IFNγ mRNA than Th17/Th22 cells similar to the ex vivo expression pattern. The expression pattern of *TBX21* after culture matched the expression of IFNγ, with the highest levels in Th17.1 and DP cells and lowest in Th17/Th22 and DN cells (Fig. 1B, lower panel). The findings on the cytokine expression pattern on the mRNA level were also found on the protein level, both in the percentage of cytokine-expressing cells (Fig. 1C, Additional file 5) and cytokine levels in the culture supernatant (Fig. 1D). These data suggest that the 3-day culture and stimulation of the sorted CCR6^+^ memTh subpopulations does not significantly alter the profile of the cells based on expression of IL-17A, IFNγ, *RORC,* and *TBX21*.

**Fig. 2:**
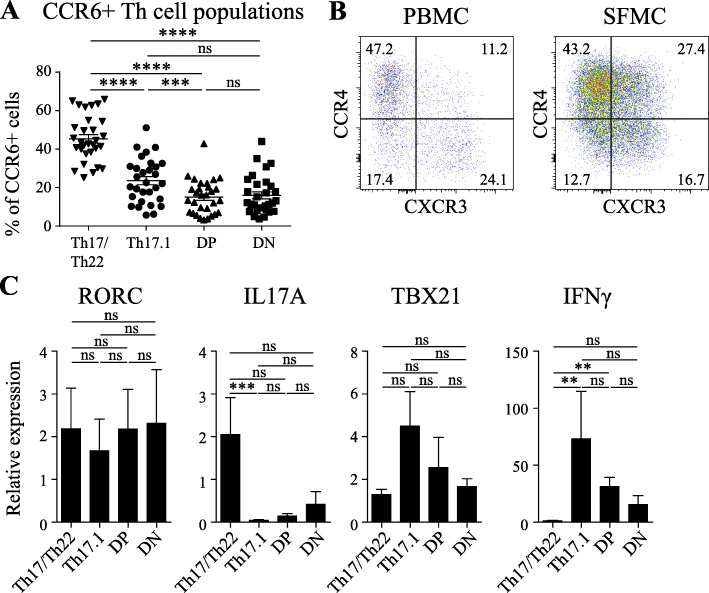
All CCR6+ memTh subpopulations are detected in RA PBMC and synovial fluid. **a** Percentage of Th17, Th17.1, DP, and DN cells in 32 treatment-naïve early RA patients. **b** Representative flow cytometric plot of CCR6+ memTh subpopulations in paired PBMC and SFMC from an established RA patient. **c** Gene transcription levels after 3 days of culture upon stimulation of anti-CD3 and anti-CD28 as measured by RT-PCR, representing n = 5–10 treatment-naïve early RA patients (mean ± SEM). **p < 0.01, ***p < 0.001, ****p < 0.0001

Although these data provide information on the healthy properties of the subpopulations, they do not necessarily represent the same phenotype as the subpopulations in inflammatory arthritis where the T cells are abnormally activated. Therefore, the CCR6^+^ subpopulations were also studied in PBMC from treatment-naïve RA patients. Similar to healthy donors, all four subpopulations were present in the PBMC of these treatment-naïve patients, with the majority being Th17/Th22 cells (Fig. 2A). Importantly, the subpopulations could also all be distinguished within the synovial fluid mononuclear cells (SFMC) and PBMC of established RA patients (Fig. 2B). Similar to the findings in healthy cells, all CCR6+ memTh subpopulations expressed equal levels of *RORC*, whereas IL-17A was predominantly produced by Th17/Th22 cells. Also, the expression patterns for IFNγ and *TBX21* were similar to the healthy situation, with the highest levels in Th17.1 cells and the lowest in Th17/Th22 cells. These data demonstrate that although all CCR6+ memTh subpopulations express equal levels of *RORC*, they differ in other parameters such as IL-17A and IFNγ. These properties are similar between healthy donors and treatment-naïve early RA patients.

### All CCR6+ subpopulations can activate synovial fibroblasts to create a pro-inflammatory feedforward loop

**Fig. 3:**
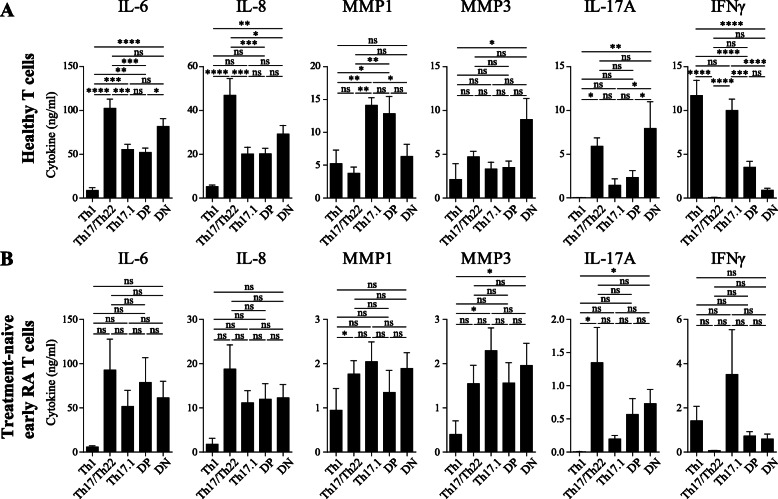
All CCR6+ subpopulation from healthy controls and RA patients activate SF. Sorted CCR6+ subpopulations from healthy donors (**A**, 25,000 T cells) or treatment-naïve early RA patients (**B**, 4000–10,000 T cells) were cultured for 3 days together with SF upon stimulation with anti-CD3 and anti-CD28. Cytokines were measured in the culture supernatant on day 3 using ELISA. Data are shown as mean ± SEM of n = 6 healthy controls or n = 5 RA patients and representative of at least 2 independent experiments. *p < 0.05, **p < 0.01, ***p < 0.001, ****p < 0.0001

To determine whether the differences in pro-inflammatory cytokines secreted by the individual CCR6+ memTh subpopulations resulted in different pathogenic potential, the ability of the subpopulations to activate synovial fibroblasts (SF) from patients with rheumatoid arthritis was investigated. CCR6+ memTh subpopulations, and also the classical Th1 cells (CCR6- CXCR3+ CCR4-) as a negative control, were sorted from healthy donors as in Fig. 1A and subsequently activated and cocultured with SF for three days, following our well-established model for synovial activation [10]. When compared to Th1 cells, all CCR6+ subpopulations activated SF as measured by increased IL-6 and IL-8 production (Fig. 3A). However, there were differences in the extent of activation, with Th17/Th22 cells being more potent inducers of IL-6 and IL-8 than Th17.1 and DP cells. Interestingly, only Th17.1 and DP cells induced MMP1 (Fig. 3A). The properties of the CCR6+ memTh populations in terms of IL-17A and IFNγ expression did not differ in the coculture with SF compared to the activated CCR6+ memTh populations only cultures in Fig. 1D, although the overall levels were slightly elevated due to the interaction with SF.

Similar to the CCR6+ memTh subpopulations from healthy individuals, those from treatment-naïve early RA patients also all activated SF upon coculture as demonstrated by the increased levels of IL-6 and IL-8, although this did not reach statistical significance (Fig. 3B). Interestingly, in contrast to the “healthy CCR6+ T cells”, the CCR6+ memTh subpopulations from RA patients did not differ in their capacity to induce IL-6 and IL-8. Furthermore, where in the healthy situation mainly Th17.1 and DP cells induce MMP1, all subpopulations from RA patients demonstrated a trend towards induction of MMP1 in SF. Similar to healthy individuals, the properties of the subsets in terms of IL-17A and IFNγ expression did not drastically change upon coculture with SF, with Th17/Th22 the highest in IL-17A and the lowest in IFNγ, in contrast to Th17.1 (Fig. 2 and 3B).

Altogether, these data indicate that all CCR6+ memTh subpopulations from both healthy individuals and RA patients can activate SF. However, there are subtle differences between the healthy and arthritic T cells in terms of which pro-inflammatory factors they predominantly activate in RASF.

### All CCR6+ subpopulations use IL-17A to induce IL-6 production by SF

**Fig. 4:**
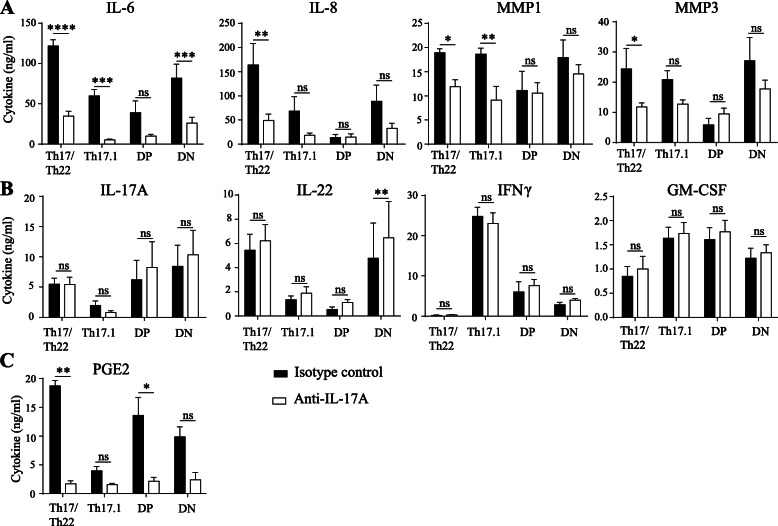
IL-17A plays a role in SF activation, but not in induction of a pro-inflammatory loop. Th17, Th17.1, DP, and DN CCR6+ memTh cells were sorted from healthy controls and cultured for 3 days with anti-CD3 and anti-CD28 stimulation, with the addition of 100 μg/ml secukinumab or an isotype control. ELISA was used to measure SF-derived mediators (**A**), T-cell derived cytokines (**B**), and PGE2 (**C**) in the culture supernatant on day 3. Data represent mean ± SEM from n = 6 subjects, pooled from 2 independent experiments. *p < 0.05, **p < 0.01, ***p < 0.001, ****p < 0.0001

Since all CCR6^+^ subpopulations are more potent activators of SF than Th1 cells despite their differences in cytokine and transcription factor expression, we next studied which factors play a role in this pro-inflammatory feedforward loop. Previously, we have shown that induction of the pro-inflammatory loop by CCR6+ Th memory cells is mediated by IL-17A [10]. However, since not all CCR6+ subpopulations express equal amounts of IL-17A, we first investigated the importance of IL-17A for SF activation in all individual CCR6+ subpopulations. Given the similar properties of the T cell subpopulations from healthy donors and RA patients and the limited availability of cells from RA patients, these functional studies were conducted with healthy donor T cells. Upon treatment with anti-IL-17A the induction of IL-6 by all subpopulations decreased, although this did not reach statistical significance for DP cells (Fig. 4A). IL-8, MMP1, and MMP3 also showed a decreasing trend upon IL-17A neutralization. The SF activation by DP cells was hardly affected by IL-17A neutralization, suggesting that these cells work via a different mechanism.

One of the features of the pro-inflammatory loop between SF and CCR6+ memTh is that increased activation of the SF leads to increased activation of the Th cells. Interestingly, despite the reduced SF activation upon IL-17A blockade, the T cell derived cytokines IL-17A, IL-22, IFNγ, and GM-CSF were not affected by anti-IL-17A treatment (Fig. 4B). Since IL-17A induction by SF depends on PGE2 [17], this lack of effect could be due to non-responsiveness of PGE2 on the anti-IL-17A treatment. However, PGE2 was significantly inhibited by anti-IL17A in Th17 and DP cells and showed a trend towards reduction in DN and Th17.1 cells (Fig. 4C). From these data, we conclude that IL-17A blockade reduces SF activation by reducing IL-6 in particular, but that these SF are still capable of further activating the Th cells. This suggests that a mechanism that does not include PGE2 or IL-6 is responsible for the Th cell activation in the SF-Th cell cocultures.

### Activation of SF by CCR6+ subpopulations is independent of IFNγ and *TBX21*

**Fig. 5:**
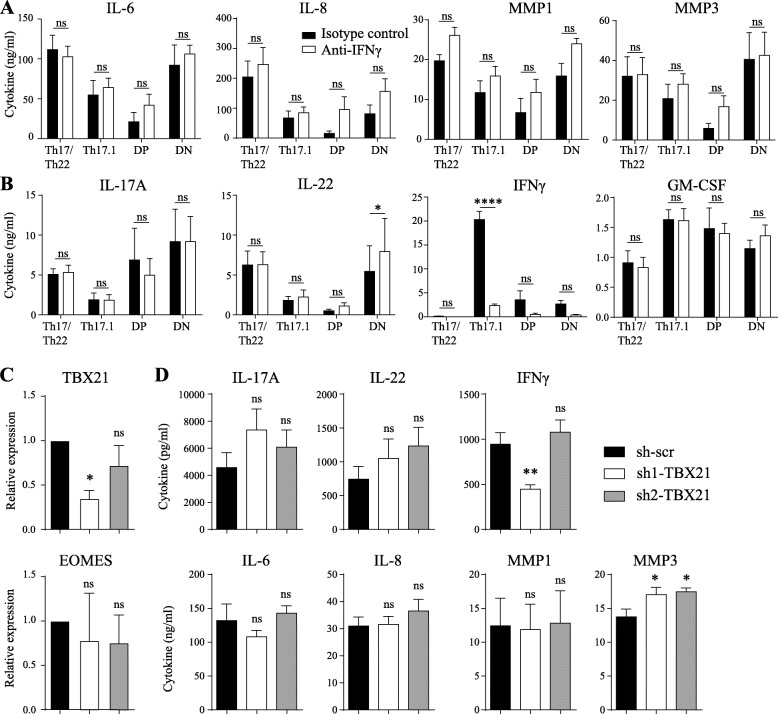
SF activation is independent of IFNγ and *TBX21*. **a**, **b** CCR6+ subpopulations were cultured and analyzed as in Fig. 4, but with 10 μg/ml neutralizing IFNγ antibody or isotype control. **c** Total CCR6+ Th memory cells were transduced with a scrambled shRNA (sh-scr) or two different *TBX21* shRNA (sh1-TBX21 and sh2-TBX21). Stably transduced CCR6+ Th memory cells were cultured for 3 days with SF under stimulation of anti-CD3 and anti-CD28. Expression of *TBX21* and *EOMES* was measured using RT-PCR on day 3. **d** Cytokine expression in culture supernatant from **c** was determined after 3 days using ELISA. Data represent mean ± SEM from n = 3–6 healthy controls, pooled from 2 independent experiments. *p < 0.05, **p < 0.01, ****p < 0.0001. In **c** and **d**, single asterisk, double asterisks, and triple asterisks represent differences compared with scrambled shRNA

Since IL-17A was mainly responsible for the induction of IL-6 by SF, other cytokines may also play a role in the pro-inflammatory loop between CCR6+ subpopulations and SF. Previous studies have shown that IFNγ suppresses production of MMP1 and MMP3 in IL-1β-stimulated SF, suggesting a modulatory role for this cytokine in the early stages of inflammation [18]. Therefore, the role of IFNγ in our system was studied. In contrast to IL-17A blockade, neutralizing IFNγ had no significant effect on the production of IL-6, IL-8, MMP1, and MMP3 (Fig. 5A). Even though less IFNγ was detected in the culture supernatant, there was no effect on the other T-cell derived cytokines (Fig. 5B).

Next to IFNγ, also *TBX21* showed a distinct pattern of expression between the subsets. Therefore, *TBX21* was knocked down using lentiviral shRNA. Due to the low cell numbers that can be obtained for the individual subpopulations, we used the entire CCR6+ Th memory pool for these experiments. In the transduced CCR6+ Th memory cells *TBX21* expression was decreased by 65% (Fig. 5C). Importantly, this repression did not induce *EOMES*, a transcription factor known to take over the role of *TBX21* (Fig. 5C). When the *TBX21*-deficient CCR6+ Th memory cells were cultured together with SF, there was no reduction in fibroblast activation as demonstrated by IL-6, IL-8, MMP1, and MMP3 expression, despite a reduction in IFNγ and a slight increase in IL-17A (Fig. 5D). Altogether, these data indicate that the activation of SF and induction of the pro-inflammatory loop between SF and CCR6+ Th memory cells are independent of IFNγ and *TBX21*.

### Both SF activation and the pro-inflammatory loop between CCR6+ subpopulations and SF can be inhibited by 1,25(OH)_2_D_3_

Previously, we have shown that blocking IL-22 does not affect the pro-inflammatory loop between CCR6+ memTh cells and SF [19], and here we found that IFNγ neutralization has no effect, while IL-17A is partially responsible for SF activation. However, the combined blockade of all pro-inflammatory T cell cytokines may have additional benefits. We have previously shown that CCR6+ memTh cells are highly susceptible to modulation by the active vitamin D metabolite 1,25(OH)_2_D_3_ [20–22], leading to decreased production of IL-17A, IL-22 and IFNγ [21, 23, 24]. Therefore, this may be a valuable strategy to block these cytokines together and evaluate the effects on SF activation.

**Fig. 6:**
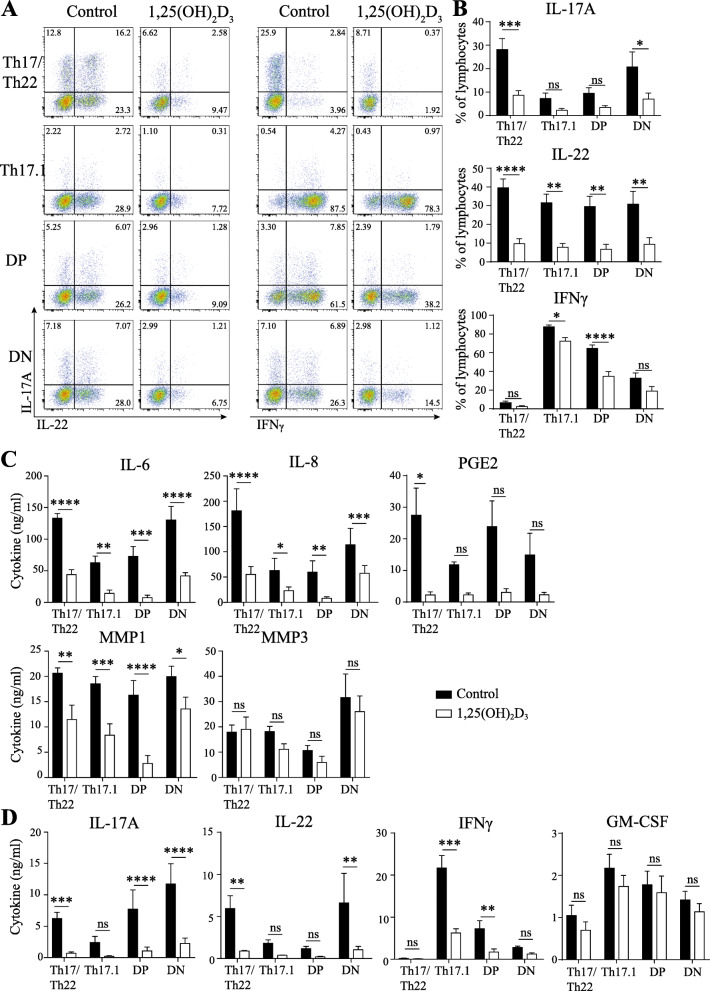
1,25(OH)_2_D_3_ can reduce SF activation and the pro-inflammatory loop. CCR6+ subpopulations were cultured with SF for 3 days with stimulation from anti-CD3 and anti-CD28 in the presence of 100 nM 1,25(OH)_2_D_3_. **a**, **b** Representative flow cytometry plots and quantification of 6 healthy donors displaying IL-17A, IL-22, and IFNγ-producing cells after 3 days of culture. **c**, **d** Expression levels of cytokines, MMPs, and PGE2 in culture supernatant on day 3. Data show mean ± SEM from n = 6 healthy controls, representative of at least 3 independent experiments. *p < 0.05, **p < 0.01, ***p < 0.001, ****p < 0.0001

Since it is unknown whether the individual CCR6+ memTh subpopulations are susceptible to 1,25(OH)_2_D_3_-mediated modulation, we first studied its effects in cultures of individual CCR6+ memTh subpopulations. As expected, the percentage of IL-17A-producing cells decreased in both Th17/Th22 cells and DN cells, the cell types that produce the highest levels of IL-17A. IL-22 was decreased by 1,25(OH)_2_D_3_ in all subpopulations, and IFNγ in Th17.1 and DP cells (Fig. 6A, B). Since this suggests that all subpopulations can be affected by 1,25(OH)_2_D_3_, the compound was added to cocultures of SF and CCR6+ memTh subpopulations. In these cultures, 1,25(OH)_2_D_3_ inhibited SF activation by all individual CCR6+ subpopulations, as demonstrated by a decrease in IL-6, IL-8, PGE2, and MMP1, but not MMP3 (Fig. 6C). 1,25(OH)_2_D_3_ also decreased the production of IL-17A, IL-22, and IFNγ similar to the data in cultures of T cells alone, but not GM-CSF (Fig. 6D). Therefore, we concluded that 1,25(OH)_2_D_3_ significantly inhibits the pro-inflammatory loop of all the individual CCR6+ memTh subpopulations cocultured with SF.

## Discussion

In this study we have demonstrated that Th17/Th22, Th17.1, DP, and DN CCR6+ memTh cells, differentiated based on CCR4 and CXCR3 expression, are individually capable of activating SF and establishing a pro-inflammatory loop which may underlie chronic synovial inflammation. This can be partly suppressed by blocking IL-17A, but not IFNγ or *TBX21*, and more effective with the multi-cytokine inhibitor 1,25(OH)_2_D_3_.

In recent years, there have been numerous studies into Th17 cell subpopulations, including their role in normal immunity and their potential pathogenicity. This led to the discovery that while IL-17A^+^ T cells can be derived directly in vitro through priming them using autologous monocytes pulsed with *Staphylococcus aureus*, IL-17A^+^IFNγ^+^ cells (also named Th17/1 cells) require *Candida albicans* [25]. Furthermore, IL-17A^+^IFNγ^+^ cells can also arise when IL-17A^+^ cells are stimulated with IL-12 and upon exposure to synovial fluid from juvenile idiopathic arthritis (JIA) patients [6, 13, 26]. However, for IFNγ^+^IL-17A^−^*RORC*^+^ (Th17.1) cells and other Th17 cell subpopulations as described in the present study, this ontogeny is less clearly defined. Since the current study shows that based on cytokine and transcription factor expression DN and DP have characteristics in between those of Th17 and Th17.1 cells, it is possible that they represent intermediate cell types that are influenced by local inflammatory conditions. To further characterize these differences and determine whether DN and DP are indeed intermediate cell types or are distinct from Th17 and Th17.1 cells, full transcriptomic and proteomic analysis are required.

In various autoimmune diseases such as multiple sclerosis, sarcoidosis, Crohn’s disease, and RA, IFNγ-producing Th17.1 cells are enriched at the site of inflammation and are thought to be especially pathogenic and drug resistant [9, 11, 14, 27, 28]. Also in juvenile idiopathic arthritis (JIA) these cells are increased in the synovial fluid compared to the peripheral blood [13, 26]. However, the present study did not imply a particular pathogenic or regulatory role of Th17.1 cells when it comes to SF activation. Our data suggest that all CCR6+ memTh subpopulations can activate SF, although there are differences between the subpopulations and between healthy individuals and patients with arthritis. Importantly, all subpopulations are present in the synovial fluid of patients with inflammatory arthritis where they can contribute to synovial inflammation. Therefore, targeting all subpopulations may be more relevant in the treatment of patients with persistent synovial inflammation than solely focusing on Th17.1 cells.

This is further supported by our finding that neutralization of IFNγ had no effect on the interaction between SF and CCR6+ memTh subpopulations. This is in contrast with previous findings that IFNγ suppresses MMP1 and MMP3 upon IL-1β stimulation [18]. This may be an effect specific for IL-1β-induced MMP production, since the interaction between CCR6+ memTh cells is largely independent of IL-1β [17], but could also be because the interplay between two cell types is more complex than just cytokine-mediated stimulation of one cell type. Interestingly, IFNγ has even been implicated to be protective in autoimmune inflammation and collagen-induced arthritis [29]. The data in this study suggest that this protective effect of IFNγ is not mediated via inhibition of fibroblast activation, since neutralization did not aggravate the inflammatory response. Overall, our data show that Th17.1 cells are not specifically pathogenic in terms of SF activation via IFNγ. Although the role of GM-CSF was not investigated in the present study, the finding that it was not inhibited by 1,25(OH)_2_D_3_ whereas other inflammatory mediators were inhibited suggests a limited role in regulating IL-6, IL-8, and MMP1 in SF. A potential effect of GM-CSF on MMP3 needs further investigation. However, in contrast to SF activation, IFNγ and GM-CSF are able to perpetuate inflammation through monocyte activation [30, 31].

Interestingly, we found that IL-17A is involved in activating RASF in all CCR6+ memTh subpopulations, even in Th17.1 cells which express very low level of this cytokine. However, only neutralizing IL-17A did not break the pro-inflammatory feedforward loop, which could be an explanation for the limited efficacy of anti-IL-17A therapy in RA patients [32–34]. Given the higher efficacy of anti-IL-17A in psoriatic arthritis [35–39], it would be of great interest to study differences in the induction of the pro-inflammatory feedforward loop by the CCR6+ memTh subpopulations between RA and PsA patients in future studies.

Previously, we have established that IL-17A, TNFα, and PGE2 all play a role in the pro-inflammatory loop between SF and CCR6+ memTh populations [17, 21, 24]. However, since none of these completely block the interaction, there must be another mechanism through which SF activate T cells aside from soluble factors. Since adhesion molecules such as CD49b, VCAM-1, and ICAM-1 have been shown to be important in T cell adherence and interaction with synovial fibroblasts [40–42], it is possible that these play an important role in the pro-inflammatory feedforward loop in synovial inflammation. Further research is required to study these mechanisms.

Our research suggests that all CCR6+ memTh cells have pathogenic potential and should be targeted to suppress synovial inflammation. This gives rise to several potential line of translational research to improve treatment in diseases driven by synovial inflammation. An interesting therapeutic option would be to utilize a broader T cell cytokine inhibitor, as demonstrated by the use of 1,25(OH)_2_D_3_ in our study. This active vitamin D metabolite has been shown to suppress Th17 pathogenicity [21, 22], and here we have shown that the individual CCR6+ memTh subpopulations are susceptible for modulation by 1,25(OH)_2_D_3_. It is possible that this higher efficacy of 1,25(OH)_2_D_3_ is due to its capacity to block multiple cytokines at once, but it could also work through blockade of cell-cell interaction via for example VCAM-1 [43, 44]. Alternatively, it has been shown that 1,25(OH)2D3 not only suppressed the pro-inflammatory capacity but also induced a regulatory phenotype in CCR6+ memTh cells, suggesting transdifferentiation that is in line with a high plasticity potential of these cells [23, 45]. Since 1,25(OH)_2_D_3_ is not a clinically applicable therapeutic due to severe side effects, the molecular mechanism underlying these changes should be investigated to find new therapeutic targets. Another possibility is the use of specific Jak inhibitors that are current being started in the treatment of inflammatory arthritis, which can also target multiple cytokine pathways together.

Finally, since all CCR6+ memTh express *RORC* and therefore by the classical definition considered Th17 cells, their pathogenicity may also be reduced by inhibiting this transcription factor. In rat models for RA pharmacological inhibition of *RORC* indeed reduced joint inflammation, suggesting it could be effective in the treatment of inflammatory arthritis [46]. However, therapeutic safety and efficacy still needs to be investigated.

## Conclusions

In conclusion, human CCR6+ memTh cells form a heterogeneous population, including Th17/Th22, DP, and Th17.1 cells that express *RORC* but differ in *TBX21*, IFNγ, and IL-17A expression. All individual human CCR6+ memTh subpopulations are more potent in activating synovial fibroblasts than classical Th1 cells in an IFNγ-independent way. Furthermore, our data suggest that IL-17A is not dominant in this T cell-RASF activation loop but that a multiple T cell cytokine inhibitor, such as 1,25(OH)2D3, but potentially also specific Jak inhibitors, is able to suppress CCR6+ memTh subpopulation-driven RASF activation. These novel data suggest that although the individual differences between the CCR6+ memTh subpopulations, there is an overlapping mechanism responsible to drive synovial activation. Therefore, future research and therapeutic interventions should focus on identifying the critical pathogenic mechanism between CCR6+ memTh subpopulations and SF interaction driving persistent synovial inflammation.

## Supplementary Information

**Additional file 1:.** Characteristics of treatment-naive early patients used in this study for PBMC.

**Additional file 2:.** Characteristics of established RA patients used in this study for RASF.

**Additional file 3:.** Primers and probes used for RT-PCR.

**Additional file 4:.** Gating strategy for sorting CCR6+ memTh subpopulations.

**Additional file 5:.** Quantification of cytokine-producing cells in Fig. 1C.

## Data Availability

Data sharing is not applicable to this article as no datasets were generated or analyzed during the current study.

## Abbreviations

ACPA: Anti-citrullinated protein antibodies
ANOVA: Analysis of variance
CCR: CC chemokine receptor
CD: Cluster of differentiation
CTLA: Cytotoxic T-lymphocyte-associated protein 4
CXCR: C-X-C motif chemokine receptor
DMEM: Dulbecco’s modified Eagle medium
DN: Double negative
DNA: Deoxyribonucleic acid
DP: Double positive
ELISA: Enzyme-linked immunosorbent assay
EOMES: Eomesodermin
FACS: Fluorescence-activated cell sorting
FCS: Fetal calf serum
GM-CSF: Granulocyte-macrophage colony-stimulating factor
HPRT: Hypoxanthine phosphoribosyltransferas
ICAM: Intercellular adhesion molecule
IFN: Interferon
IL: Interleukin
IMDM: Iscove’s modified Dulbecco’s medium
memTh: Memory T helper cells
MHC: Major histocompatibility complex
MMP: Matrix metalloproteinase
PBMC: Peripheral blood mononuclear cells
PBS: Phosphate-buffered saline
PGE2: Prostaglandin E2
PHA: Phytohaemagglutinin
PMA: Phorbol 12-myristate 13 acetate
PsA: Psoriatic arthritis
PTPN22: Protein tyrosine phosphatase, non-receptor type 22
RA: Rheumatoid arthritis
RASF: RA synovial fibroblasts
RNA: Ribonucleic acid
RORC: RAR Related Orphan Receptor C
RT-PCR: Reverse transcription polymerase chain reaction
SF: Synovial fibroblasts
SFMC: Synovial fluid mononuclear cells
shRNA: Short hairpin
TBX21: T-Box Transcription Factor 21
TNFα: Tumor necrosis factor alpha
VCAM: Vascular cell adhesion molecule
