# Supplementary material for: The heterogeneous human memory CCR6+ T helper-17 populations differ in T-bet and cytokine expression but all activate synovial fibroblasts in an IFNγ-independent manner
Source: Arthritis Res Ther. 2021 Jun 3;23:157. doi: 10.1186/s13075-021-02532-9 (PMC8173960; doi:10.1186/s13075-021-02532-9)
Supplement: Supplementary file 1 — Additional file 1:. Characteristics of treatment-naive early patients used in this study for PBMC. [file 13075_2021_2532_MOESM1_ESM.docx]

| **Parameters** | **Treatment-naïve early RA patients (n=41)** |
| --- | --- |
| Age (years), *mean (SD)* | 52.1 (13.3) |
| DAS | 3.3 (2.9-3.8) |
| Female gender, *% (n)* | 68.3 (28) |
| RF positive, *% (n)* | 63.4 (26) |
| ACPA positive, *% (n)* | 73.2 (30) |
| VAS global, *mean (SD)* | 55.0 (22.3) |
| ESR | 23 (15-38.5) |
| CRP | 9 (5-18) |
| TJC44 | 11 (5-16) |
| SJC44 | 6 (4-9.5) |
| Duration of complaints (days), *mean (SD)* | 158.5 (85.1) |

**Characteristics of treatment-naive early patients used in this study for PBMC.**
Data are reported as median (IQR) unless otherwise indicated. Abbreviations: DAS, disease activity score; RF, rheumatoid factor; ACPA, anti-citrullinated peptide antibodies; VAS, visual analogue scale; ESR, erythrocyte sedimentation rate; CRP, c-reactive protein; TJC, tender joint count; SJC, swollen joint count.
