# Supplementary material for: The heterogeneous human memory CCR6+ T helper-17 populations differ in T-bet and cytokine expression but all activate synovial fibroblasts in an IFNγ-independent manner
Source: Arthritis Res Ther. 2021 Jun 3;23:157. doi: 10.1186/s13075-021-02532-9 (PMC8173960; doi:10.1186/s13075-021-02532-9)
Supplement: Supplementary file 2 — Additional file 2:. Characteristics of established RA patients used in this study for RASF. [file 13075_2021_2532_MOESM2_ESM.docx]

| **Parameters** | **Established RA patients (n=6)** |
| --- | --- |
| Age (years), *mean (SD)* | 62.5 (17.6) |
| Female gender, *% (n)* | 66.7 (4) |
| RF positive, *% (n)* | 60 (3)* |
| ACPA positive, *% (n)* | 40 (2)* |
| Duration of complaints (years), *mean (SD)* | 13.6 (5.9)* |

**Characteristics of established RA patients used in this study for RASF.**
Abbreviations: RF, rheumatoid factor; ACPA, anti-citrullinated peptide antibodies; *, 1 patient data not recorded
