# Supplementary material for: The heterogeneous human memory CCR6+ T helper-17 populations differ in T-bet and cytokine expression but all activate synovial fibroblasts in an IFNγ-independent manner
Source: Arthritis Res Ther. 2021 Jun 3;23:157. doi: 10.1186/s13075-021-02532-9 (PMC8173960; doi:10.1186/s13075-021-02532-9)
Supplement: Supplementary file 3 — Additional file 3:. Primers and probes used for RT-PCR. [file 13075_2021_2532_MOESM3_ESM.docx]

| **Gene** | **Forward primer** | **Reverse primer** | **Probe #** |
| --- | --- | --- | --- |
| HPRT | 5’-TGACCTTGATTTATTTTGCATACC-3’ | 5’-CGAGCAAGACGTTCAGTCCT-3’ | 73 |
| IL17A | 5’-TGGGAAGACCTCATTGGTGT-3’ | 5’-GGATTTCGTGGGATTGTGAT-3’ | 8 |
| IL22 | 5’-CAACAGGCTAAGCACATGTCA-3’ | 5’-ACTGTGTCCTTCAGCTTTTGC-3’ | 6 |
| IFNG | 5’-GGCATTTTGAAGAATTGGAAAG-3’ | 5’-TTTGGATGCTCTGGTCATCTT-3’ | 21 |
| GMCSF | 5’-TCTCAGAAATGTTTGACCTCCA-3’ | 5’-GCCCTTGAGCTTGGTGAG-3’ | 1 |
| RORC | 5’-CAGCGCTCCAACATCTTCT-3’ | 5’-CCACATCTCCCACATGGACT-3’ | 69 |
| TBX21 | 5’-TGTGGTCCAAGTTTAATCAGCA-3’ | 5’-TGACAGGAATGGGAACATCC-3’ | 9 |
| EOMES | 5’-CTTCTACCCGCTGGAGAGTG-3’ | 5’-TGTCTAAGTCCAACTTCTGAGGAGA-3’ | 10 |

**Primers and probes used for RT-PCR.**
