# Supplementary material for: The heterogeneous human memory CCR6+ T helper-17 populations differ in T-bet and cytokine expression but all activate synovial fibroblasts in an IFNγ-independent manner
Source: Arthritis Res Ther. 2021 Jun 3;23:157. doi: 10.1186/s13075-021-02532-9 (PMC8173960; doi:10.1186/s13075-021-02532-9)
Supplement: Supplementary file 4 — Additional file 4:. Gating strategy for sorting CCR6+ memTh subpopulations. [file 13075_2021_2532_MOESM4_ESM.docx]

**
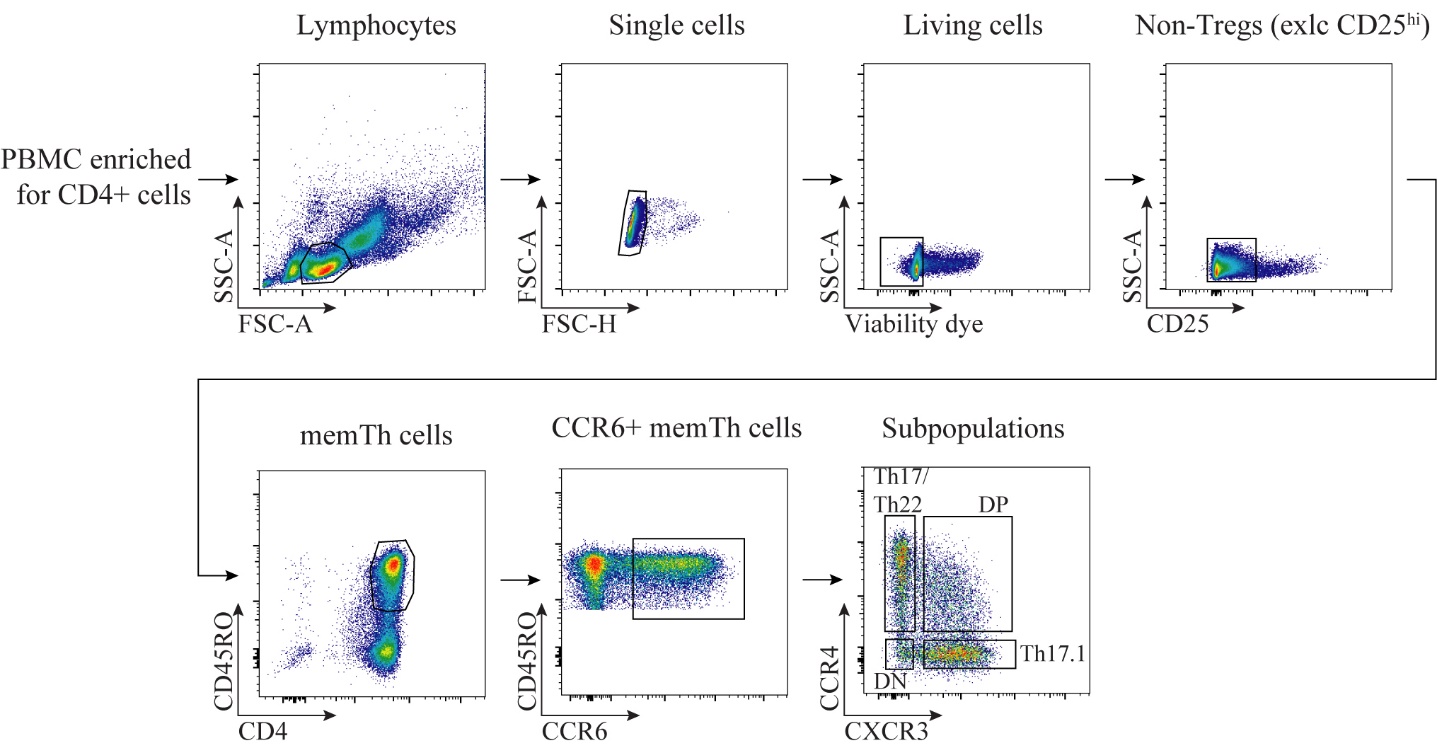
**

**Gating strategy for sorting CCR6+ memTh subpopulations.**

PBMC were pre-sorted for CD4+ using MACS. Then the above outlined gating strategy was followed to isolated single living lymphocytes, that were not Tregs (through CD25^hi^ exclusion) and were positive for CD4, CD45RO and CCR6. Then the subpopulations were isolated based on CCR4 and CXCR3 expression.
