# Supplementary material for: The heterogeneous human memory CCR6+ T helper-17 populations differ in T-bet and cytokine expression but all activate synovial fibroblasts in an IFNγ-independent manner
Source: Arthritis Res Ther. 2021 Jun 3;23:157. doi: 10.1186/s13075-021-02532-9 (PMC8173960; doi:10.1186/s13075-021-02532-9)
Supplement: Supplementary file 5 — Additional file 5:. Quantification of cytokine-producing cells in Fig. 1C. [file 13075_2021_2532_MOESM5_ESM.docx]

**
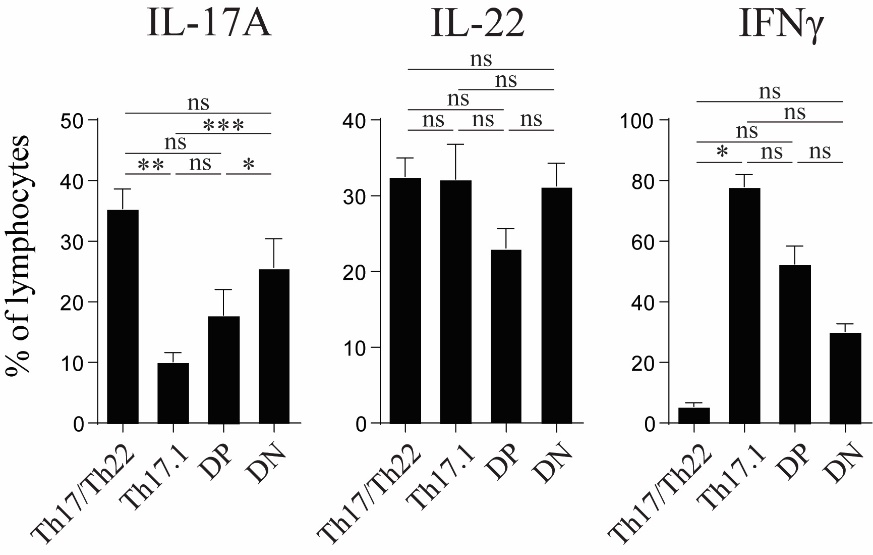
**

**Quantification of cytokine-producing cells in figure 1C.**

CCR6+ memTh subpopulations were sorted and stimulated with anti-CD3 and anti-CD28 for three days. Data represent mean ± SEM of 3-6 donors, representative of at least 2 independent experiments. *p<0.05, **p<0.01, ***p<0.001.
